# Industrial robots and carbon emission reductions in global value chains

**DOI:** 10.1016/j.isci.2026.117024

**Published:** 2026-08-11

**Authors:** Genqiang Li, Lingmeng Kong

**Affiliations:** 1 School of Humanities and Social Sciences, Beihang University, Beijing 100191, China

**Keywords:** industrial robots, carbon emission intensity, global value chain, green total factor productivity, energy consumption

## Abstract

As a representative outcome of digital technological advancement, the use of industrial robots (IRs) is a key pathway toward economic transformation and green development. By leveraging global industry-level data, this paper examines the impact of IRs on carbon emission intensity (CEI) through the lens of global value chains (GVCs). The results demonstrate that IRs significantly suppress the CEI. This decarbonization effect is particularly pronounced in economically developed regions, within secondary and tertiary industries, and in labor- and capital-intensive sectors. In terms of impact mechanisms, IRs curtail CEI primarily by increasing green total factor productivity (GTFP), scaling down energy consumption, and optimizing energy structure. Furthermore, from the perspective of GVCs, higher GVC participation, forward GVC participation pattern, and elevated GVC position all strengthen IRs’ decarbonization effect. This study offers policy-relevant insights for achieving carbon peaking and carbon neutrality goals.

## Highlights

• Industrial robots (IRs) significantly suppress carbon emission intensity (CEI)
• The effects are stronger in developed regions and labor/capital-intensive sectors
• IRs reduce CEI mainly through GTFP, energy consumption, and energy structure
• The GVC degree, pattern, and position amplify the carbon reduction effect of IRs

## Graphical abstract

**Figure:**
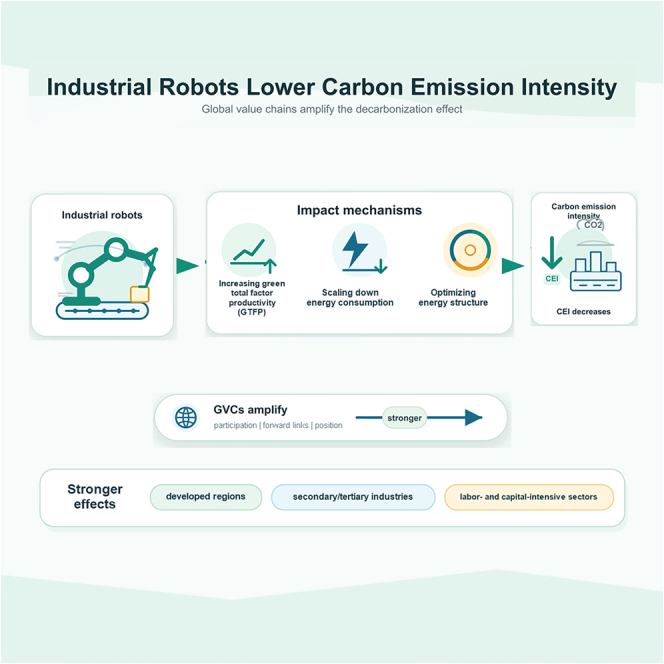

## Teaser

Environmental science; Energy engineering; Industrial engineering

## Introduction

Greenhouse gas emissions have raised global temperatures and increased the occurrences of extreme weather events, including sea level rise and ocean acidification, all of which have significant socio-economic consequences.^1^ With a 66% contribution to the warming impact, carbon dioxide ranks as the most significant greenhouse gas in the atmosphere according to the World Meteorological Organization. As a strategic emerging industry, industrial robots (IRs) are pivotal for driving economic transformation and achieving carbon neutrality.^2^ The International Federation of Robotics (IFR) noted that in 2022, there were 553,052 IR installations in factories around the world, representing a 5% increase annually. As nations strive for carbon neutrality, the role of strategic technologies in reconciling economic growth with environmental sustainability becomes paramount. IRs, a cornerstone of the Industry 4.0 paradigm centered on automation, connectivity, and data-driven efficiency, are at the forefront of this transformation. Therefore, investigating the effect of IRs on the carbon emission intensity (CEI) holds significant practical value in realizing carbon neutrality objectives. Moreover, the international division of production has developed into the global value chain (GVC) division, and owing to the heterogeneous characteristics of countries and industries, such as degree of participation, pattern, and position in the GVC division, the carbon reduction effect of IRs may be affected from the perspective of global industry linkages.

The International Organization for Standardization (ISO) defines robots as “reprogrammable, multipurpose, automatically controlled manipulators” for use in industrial automation applications, which can be tools and machinery that are programmed using artificial intelligence technology to carry out a variety of manufacturing tasks.^3^ Existing studies can be categorized into the following two main views: first, the application of robots can reduce pollution emissions from the production process and promote the development of a green economy^4^^,^^5^; second, the environmental rebound effect suggests that efficiency gains may stimulate expansion in output and energy consumption, partially or fully offsetting the environmental benefits.^6^^,^^7^ Accordingly, the net effect of IRs on carbon emissions and the underlying transmission mechanisms remain to be further clarified.

Theoretically and methodologically, GVCs are defined as cross-border production activities spanning the entire life cycle from conception to final use. The core value of GVC analysis lies in tracing value-added across borders, moving beyond traditional trade statistics to reveal a country’s or industry’s actual position in the international division of labor. GVC indicators, typically derived from global input-output tables, capture sectoral heterogeneity and cross-border production linkages.^8^^,^^9^ As production becomes increasingly fragmented, environmental performance is shaped not only by domestic capabilities but also by a country’s position and mode of integration into GVCs. Recent research, particularly on the automotive sector, shows that GVC governance influences environmental outcomes: lead firms set standards, while lower-tier suppliers may face pressures for green upgrading (the process by which an enterprise or economy adopts environmentally friendly processes, technologies, and management practices in GVC to improve its environmental performance while improving its economic status) or be pushed into a “race to the bottom.”^10^^,^^11^^,^^12^ Meanwhile, digital technologies—especially the recent proliferation of IRs—have become key drivers of industrial transformation and value chain upgrading.^2^ In the context of economic globalization, pollution transfers have also drawn scholarly attention. Some researchers have integrated GVCs and carbon emissions into a unified framework, arguing that the participation in GVCs enables technological progress and environmental improvement through technology spillovers and regulatory pressures.^13^^,^^14^

This study argues that the carbon reduction effect of IRs is not automatic but is critically moderated by GVC structural characteristics. While extensive literature exists on the determinants of carbon emissions and the economic effects of robotic application, certain aspects of this research landscape could be further developed. First, most environmental studies on robots have focused on general pollution or air quality, rather than the direct relationship between robots and carbon emissions. Second, research on how technological progress affects carbon intensity often relies on production-side CEI indicators, which fail to account for inter-industry linkages and embodied carbon emissions computed via input-output tables. In contrast, our study employs the aggregate embodied intensity (AEI) as the metric, enabling a more accurate assessment of CEI.

This paper’s primary innovations and contributions are 3-fold. First, adopting a GVC lens, it incorporates GVC structural features—participation degree, participation pattern, and position—to elucidate the role of GVC in enhancing the emission-reducing effects of IRs. Moreover, the use of input-output tables to calculate a more accurate CEI indicator, which accounts for embodied carbon in trade, enables us to move beyond a narrow, territorial accounting lens. Second, the study delineates the mechanism through which IRs affect the CEI, specifically via green total factor productivity (GTFP), the energy consumption scale, and energy structure optimization (which refers to the transformation process of reducing the proportion of fossil energy, especially coal, and increasing the proportion of renewable energy and clean electricity in total energy consumption). Third, it identifies and tests three transmission channels: GTFP, energy consumption scale, and energy structure. It also conducts cross-country and cross-industry heterogeneity analyses, providing policy implications for achieving carbon peaking and carbon neutrality.

This study regards IRs as a core technology of Industry 4.0. Although Industry 5.0 emphasizes human centricity, systemic resilience, and circularity,^15^ the findings of this study remain highly relevant: robots provide the automation foundation on which human-machine collaboration in Industry 5.0 is built. We position IRs as transitional infrastructure rather than a final technological destination. Their carbon reduction performance can provide empirical insights for integrating legacy automation into future sustainability-oriented socio-technical systems.

## Results

### Descriptive statistics of the variables

Descriptive statistics for key variables are reported in Table 1. Table 1 shows that the standard deviation of the variable ln*Robot* is relatively large, indicating that there are obvious differences in the annual stock of robots in various industries across countries. The scale of IRs varies depending on the characteristics of the industry and the stage of development of the country, which, in turn, has different effects on CEI.

**Table 1:** Descriptive statistics of the variables

Variable	*n*	Mean	SD	Min	p50	Max
lnAEI	16,716	−0.655	0.854	−3.532	−0.763	1.700
lnRobot	16,716	−4.340	7.664	−11.510	−11.510	9.387
lnpgo	16,716	5.854	2.025	2.639	5.361	12.940
lnkl	16,716	5.600	2.178	2.180	5.022	13.370
urb	16,716	71.540	13.760	29.570	73.640	97.560
RD	16,716	1.482	0.896	0.237	1.262	3.705
GTFP	15,458	1.073	0.180	0.664	1.008	1.877
pec	15,855	5.287	1.857	0.055	4.932	10.190
struct	16,281	0.291	0.236	0.000	0.260	0.862
lnGVC_par	16,664	3.706	0.403	2.225	3.784	4.351
lnGVC_f	16,664	3.111	0.654	0.859	3.236	4.190
lnGVC_b	16,556	2.642	0.702	0.229	2.803	3.743
lnGVC_pos	16,716	−0.030	0.107	−0.311	−0.024	0.257

### Benchmark regression results

Table 2 presents the regression results for the impact of IRs on CEI. The coefficients of ln*Robot* are consistently negative and statistically significant at the 1% level regardless of the addition of control variables or fixed effects (FEs), indicating that the CEI decreases as the IRs increase. Hypothesis 1 is, therefore, verified. The adoption of IRs optimizes production processes and enables intelligent manufacturing practices, including precision energy control and predictive maintenance,^16^ thereby enhancing resource and energy efficiency. This reduces both the resource intensity and the carbon intensity of output.

**Table 2:** Benchmark regression

Variables	(1)	(2)	(3)	(4)
	lnAEI	lnAEI	lnAEI	lnAEI
lnRobot	−0.028∗∗∗	−0.033∗∗∗	−0.002∗∗∗	−0.002∗∗∗
	(-35.29)	(-41.78)	(-2.87)	(-2.87)
lnpgo		0.157∗∗∗	0.078∗∗∗	0.079∗∗∗
		(20.09)	(6.40)	(6.43)
lnkl		−0.069∗∗∗	−0.036∗∗∗	−0.036∗∗∗
		(-9.06)	(-5.55)	(-5.58)
urb				0.007∗∗∗
				(2.78)
RD				−0.033∗∗
				(-2.01)
Constant	−0.777∗∗∗	−1.333∗∗∗	−0.918∗∗∗	−1.379∗∗∗
	(-113.08)	(-71.50)	(-14.61)	(-7.05)
Industry FE	NO	NO	YES	YES
Country FE	NO	NO	YES	YES
Year FE	NO	NO	YES	YES
Observations	16,716	16,716	16,716	16,716
R^2^	0.063	0.110	0.796	0.797

Note: All values in parentheses are robust t statistics; ∗, ∗∗, and ∗∗∗ indicate significance at the 10%, 5%, and 1% levels, respectively. Subsequent tables use the same notation..

According to the data in column (4) of Table 2, the coefficients of ln*pgo* and *urb* are both significantly positive, demonstrating that they increase the overall energy demand, which, in turn, increases the CEI and deteriorates the ecological environment. The coefficients of ln*kl* and *RD* suggest that the shift from an extensive to an intensive mode of production through the transformation of the mode of economic development, the development of green and energy-saving technologies, and the implementation of energy-saving and emission reduction facilities will be effective in curbing CEI.

### Heterogeneity analysis

#### By economic development level

To explore whether the level of economic development affects the carbon reduction effect of IRs, the 42 selected countries/regions are categorized as developed or developing economies on the basis of International Monetary Fund (IMF) criteria. The classification is available to the readers upon request.

As shown in Table 3, for developed economies, the impact coefficient of IRs was significant, at −0.002, while for developing economies, the impact coefficient of IRs was only −0.001 and not significant. This may be because the greater the degree of economic development is, the more complete the robot’s ecosystem is and the more favorable the soft environment for IRs is. Under the dual effect of industrial agglomeration and human capital agglomeration, the carbon reduction effect of IRs is fully utilized, whereas the opposite is true for less economically developed economies.^17^ As a result, our estimates suggest a significant negative relationship between widespread IRs and CEI in economically developed economies.

**Table 3:** Heterogeneity results: By economic development level and industry category

Variables	Economies		Industry		
	Developed	Developing	Primary	Secondary	Tertiary
	lnAEI	lnAEI	lnAEI	lnAEI	lnAEI
lnRobot	−0.002∗∗∗	−0.001	−0.002	−0.003∗∗∗	−0.007∗∗∗
	(-3.06)	(-0.89)	(-0.62)	(-4.30)	(-3.25)
CONTROL	YES	YES	YES	YES	YES
FE	YES	YES	YES	YES	YES
Observations	12,982	3,734	1,720	13,275	1,721
R^2^	0.800	0.801	0.609	0.824	0.849

Note: “YES” in the rows “CONTROL” and “FE” indicates incorporation of control variables and country-, industry-, and year-fixed effects. Subsequent tables use the same notation..

#### By industry category

As shown in Table 3, IRs have a negative inhibitory effect on the CEI for different types of industries, but the effect on the primary industry, namely agriculture, is not significant. The possible reason for this difference is that large-scale IRs have not yet been developed in agriculture, and the scale effect of IRs has not yet manifested; thus, the carbon reduction effect of IRs has not been fully exploited. The heavy industry of the secondary industry and the transportation, storage, and postal sectors of the tertiary industry are the main contributors to the CEI. IRs have a greater marginal contribution to reducing the CEI of these two industries, which provides effective technical support for realizing the green transformation and upgrading of the industries. Therefore, IRs have different carbon reduction effects on different industries, and the carbon emission reduction effects on the secondary and tertiary industries are more significant.

To further elucidate the mechanisms underlying industry heterogeneity, it is instructive to contrast two archetypal GVC governance structures. In the automotive industry, which exemplifies producer-driven GVCs characterized by capital and technology intensity, robots are deeply embedded in core production processes including welding, painting, and assembly. Their carbon reduction contribution operates primarily through enhancing production precision, reducing defect rates, and optimizing energy consumption across integrated production lines. Moreover, this emissions mitigation potential is amplified through alignment with stringent global environmental standards, such as EU emission regulations that incentivize cleaner manufacturing processes. Conversely, in the food processing industry, which typifies buyer-driven GVCs with labor and resource intensity, robotic applications are predominantly concentrated in downstream processes such as sorting, packaging, and palletizing. The carbon reduction potential in this context manifests primarily through minimizing post-harvest losses and optimizing cold chain logistics, although these efficiency gains are often constrained by the peripheral nature of automation relative to core production activities, and the overall emission mitigation effect remains more modest than in capital-intensive manufacturing sectors. This differential stems fundamentally from divergent production logics: in producer-driven chains, robots constitute the technological core of value creation and emission control, whereas in buyer-driven chains, they serve primarily as peripheral tools for the operational efficiency.

One potential alternative explanation for the observed correlation is that cleaner industries might adopt robots earlier due to pre-existing environmental preferences. However, our regression models include industry FEs, which absorb time-invariant, industry-specific factors such as inherent green orientation or technological sophistication. Thus, the estimated coefficients primarily capture within-industry variations over time. Moreover, the primary drivers of robot adoption are cost reduction and automation demands—such as substituting for labor, increasing productivity, or performing dangerous tasks—rather than environmental considerations.^3^ Nevertheless, we acknowledge that the possibility of time-varying green preferences influencing both robot adoption and CEI remains a residual endogeneity concern, which should be noted as a limitation and addressed in future research with more exogenous variation.

#### By the factor intensity of manufacturing industries

Manufacturing industries are grouped into three types on the basis of the factor intensity (the classification is available to the readers upon request): labor intensive, capital intensive, and technology intensive. As shown in Table 4, IRs have a negative effect on CEI for various types of manufacturing industries, but this effect is not statistically significant for technology-intensive industries. The development of green high-tech areas and greater demand for digital infrastructure and energy input are potential explanations. However, labor-intensive and capital-intensive sectors still rely heavily on energy and resources and continue to be the main sources of carbon emissions. By driving the transformation of labor-intensive and capital-intensive sectors into cleaner, more technology-intensive and higher value-added sectors, the application of IRs fosters the advancement of low-emission and highly efficient production systems.^18^ As a result, IRs in these two sectors have a greater effect on lowering the CEI. Additionally, because labor-intensive industries have high labor demand, manual operation without improvement tends to increase the CEI. In contrast, IRs lower carbon emissions per unit of output by increasing resource utilization; thus, IRs significantly lower CEI in labor-intensive industries.

**Table 4:** Heterogeneity results: By the factor intensity of manufacturing industries

Variables	(1)	(2)	(3)
	Labor-intensive	Capital-intensive	Technology-intensive
	lnAEI	lnAEI	lnAEI
lnRobot	−0.005∗∗∗	−0.002∗	−0.001
	(-4.46)	(-1.73)	(-0.48)
CONTROL	YES	YES	YES
FE	YES	YES	YES
Observations	3,499	7,589	2,187
R^2^	0.748	0.824	0.866

### Robustness test

#### Replacing dependent variables

The dependent variable (ln*AEI*) is replaced with the CO_2_ emission intensity provided by the World Bank (WDI) to explore the robustness of the benchmark regression results. Column (1) of Table 5 shows that the coefficient of IRs (ln*Robot*) remains stable at approximately −0.001 and is significant at the 1% level, suggesting a significant negative relationship between IRs and CEI.

**Table 5:** Robustness test for the impact of IRs on CEI

Variables	(1)	(2)	(3)	(4)	(5)	(6)
	CEI	Excluding low-polluting economies	Excluding low-polluting industries	Adding lags	Industry × country fixed effect	2SLS
lnRobot	−0.001∗∗∗	−0.003∗∗∗	−0.002∗∗∗	–	−0.002∗∗∗	−0.002∗∗∗
	(-7.49)	(-3.71)	(-2.74)	–	(-5.15)	(-2.58)
L.lnRobot	–	–	–	−0.002∗∗∗	–	–
	–	–	–	(-2.58)	–	–
CONTROL	YES	YES	YES	YES	YES	YES
Industry FE	YES	YES	YES	YES	NO	YES
Country FE	YES	YES	YES	YES	NO	YES
Year FE	YES	YES	YES	YES	YES	YES
Industry × country FE	NO	NO	NO	NO	YES	NO
Observations	16,716	12,890	12,244	15,146	16,716	15,146
R-squared	0.972	0.783	0.765	0.800	0.959	0.008

#### Excluding low-polluting countries and low-polluting industries

The ability of IRs to reduce the CEI is easily overestimated for countries and industries with low emissions because of various confounding factors and circumstances. As a result, it is challenging for the overall regression results to accurately reflect reality. For this reason, the lowest 25% of countries and industries with pollution discharge were excluded to obtain more accurate results. According to columns (2) and (3) of Table 5, our estimates suggest a significant negative relationship between IRs and CEI, and the findings are robust.

#### Adding lags

IRs installed during this period may not be immediately operational owing to limitations in related facilities and human capital. With respect to the installation and use of robots, businesses need a certain amount of time to invest in worker professional and technical training and establish management systems, production processes, and related intelligent facilities that are robot compatible. It takes time for the mechanism reducing the CEI to work. Hence, IRs may support a delay in decreasing the CEI. This paper proposes a lag term for IRs to explore the lagged influence of IRs on the CEI and test the validity of the aforementioned conclusions. In column (4) of Table 5, the stock of robots in the lag period (L.ln*Robot*) is significantly negative, at approximately −0.002, which is consistent with the baseline regression results.

#### Adjusting control methods for FEs

To further alleviate the endogeneity problem of model estimation and enhance the goodness-of-fit of the model, we set the interaction term between industry FEs and country FEs to control these unobservable factors by referring to Li and Liao.^19^ The regression coefficient of IRs is significant at −0.002 in column (5) of Table 5, thereby confirming the robustness of our baseline results.

#### Instrumental variable regression

In accordance with the two-stage least squares (2SLS) method, this study employed lagged IRs as an instrumental variable to mitigate potential endogeneity concerns. The first-stage regression demonstrates a strong correlation between the instrument and the endogenous explanatory variable. The results from under-identification tests yield a *p* value less than 0.01. Furthermore, the Cragg-Donald Wald F statistic was 35659.92, and the Kleibergen-Paap Wald rk F was 14218.45, rejecting the null hypothesis that the instrumental variables are weak. As listed in column (6) of Table 5, the coefficient of IRs remained statistically significant and negative at approximately −0.002, reinforcing the robustness of the study’s conclusions.

## Discussion

### Mechanism analysis

#### Mechanism 1: GTFP

It is clear from the previous theoretical analysis that expanding the scope of IRs will reduce energy consumption and CEI from both input and output. The mechanism path proposed in this section is as follows: As the IRs increase, the GTFP increases, and then the CEI decreases. Specifically, the expansion of IRs propels firms to intensify R&D activities focused on energy production and pollution control. This accelerates energy technology innovation and improves energy efficiency,^20^ resulting in the rise of GTFP and ultimately contributing to a reduction in CEI.

As shown in Table 6, column (1) presents the benchmark regression results. Column (2) shows a significantly positive coefficient for IRs (ln*Robot*), suggesting that IRs contribute effectively to the improvement of GTFP. With the inclusion of the mediating variable in column (3), GTFP has a significantly negative effect on the CEI. This is evidence that IRs reduce CEI by enhancing GTFP, thereby supporting hypothesis H2a.

**Table 6:** Mechanism analysis 1: GTFP

Variables	(1)	(2)	(3)
	lnAEI	GTFP	lnAEI
lnRobot	−0.002∗∗∗	0.001∗∗∗	−0.001∗∗
	(-2.87)	(2.99)	(-2.16)
GTFP	–	–	−0.264∗∗∗
	–	–	(-10.79)
CONTROL	YES	YES	YES
FE	YES	YES	YES
Observations	16,716	15,458	15,458
R^2^	0.797	0.324	0.801

#### Mechanism 2: Scale of energy consumption

IRs can contribute to reduced energy waste during production and enable more precise control over energy usage. The mechanism hypothesized in this section is that an increase in IRs will reduce per capita energy consumption, which, in turn, will reduce the CEI.

In Table 7, column (2) indicates that increasing IRs are effective at reducing the scale of energy consumption, and column (3) shows that after per capita energy consumption is added, the effect of this variable on the CEI is significantly positive. Thus, the mechanism by which IRs reduce the CEI by reducing the scale of energy consumption (H2b) is verified. CEI is highly dependent on energy consumption, whereas robots reduce redundant energy consumption and waste by realizing real-time precision control of the production process. This enhancement boosts production and the energy utilization efficiency, thereby directly influencing the environmental intensity,^21^ effectively reducing carbon emissions per unit of output and providing technical support for the improvement of the ecological environment.

**Table 7:** Mechanism analysis 2: Scale of energy consumption

Variables	(1)	(2)	(3)
	lnAEI	pec	lnAEI
lnRobot	−0.002∗∗∗	−0.004∗∗∗	−0.002∗∗
	(-2.87)	(-2.83)	(-2.50)
pec	–	–	0.214∗∗∗
	–	–	(29.75)
CONTROL	YES	YES	YES
FE	YES	YES	YES
Observations	16,716	15,855	15,855
R^2^	0.797	0.857	0.843

#### Mechanism 3: Structure of energy consumption

IRs can effectively revolutionize the types of energy needed for production by using electricity and relying on spillover effects to support the development of green technology. This will compel a structural shift in energy consumption, moving from fossil-based sources to renewable alternatives. Therefore, it is hypothesized that increasing IRs will improve the structure of energy consumption, decrease the share of fossil fuel energy in overall energy consumption, and consequently lessen the CEI.

Column (2) of Table 8 demonstrates a statistically significant negative effect of IRs on the share of fossil fuel energy consumption. When the mediating variable (energy consumption structure) is added to the baseline model, column (3) shows that its effect on CEI is significantly positive. As a result, the mechanism through which IRs reduce the CEI by improving the energy consumption structure (H2c) is verified. The utilization of IRs fosters a greener energy structure, thereby reducing the CEI and consequently exerting a positive impact on the environment.^22^ To summarize the discussion, hypotheses H2a–H2c are verified.

**Table 8:** Mechanism analysis 3: Structure of energy consumption

Variables	(1)	(2)	(3)
	lnAEI	struct	lnAEI
lnRobot	−0.002∗∗∗	−0.001∗	−0.002∗∗∗
	(-2.87)	(-1.89)	(-3.25)
struct	–	–	0.438∗∗∗
	–	–	(18.78)
CONTROL	YES	YES	YES
FE	YES	YES	YES
Observations	16,716	16,281	16,281
R^2^	0.797	0.578	0.805

### Moderating effects of GVCs

Since the beginning of the 21st century, a new wave of technological advancement has pushed the GVC to deepen. As a result, to meet the broad demands of the global market and reap the benefits of the division of labor, countries have a strong incentive to increase the value added of production by utilizing highly developed technology. Because of the continued spread of green innovation and technology components through the GVC, the level and scope of IRs in the relevant economies will change. With the increasing prosperity of foreign trade and foreign investment, on the one hand, the pattern of the international production division will change; on the other hand, local carbon emissions will cross the border, evolving into a global environmental problem. Thus, the carbon emission problem is closely related to the GVC. The effect of IRs on the CEI is also affected by the division characteristics of GVCs.

#### Moderating effect of the degree of GVC participation

An increasing degree of GVC participation indicates that a country deepens its economic and trade relations with other countries. The adoption of cutting-edge technology through borrowing and assimilation is conducive to the innovation of the country’s industrial structure, the formation of a complete robot industry chain, the breakthrough of key technologies, and the formation of the key driving force of CEI reduction. Therefore, the proposed hypothesis in this section is as follows: when the degree of GVC participation increases, the carbon reduction effect of IRs is amplified.

As illustrated in Table 9, the coefficient for the degree of GVC participation (ln*GVC_par*) is significantly positive, implying that the CEI tends to increase with greater engagement in GVCs. Moreover, the interaction term between IRs and GVC participation (ln*Robot* × ln*GVC_par*) in column (3) has a significantly negative coefficient of −0.005. This suggests that although higher GVC participation may initially increase emissions, it ultimately enhances the carbon reduction effect of IRs, indicating a synergistic role of GVC integration in boosting the green productivity gains from automation. A plausible interpretation of these findings is that they reveal a dynamic process, characteristic of a nation’s green transformation and upgrading within GVCs. In the initial phase, trade liberalization and production expansion fostered by GVC participation primarily contribute to absolute energy consumption and carbon emissions through the scale effect—that is, the increase in total economic output. Concurrently, the pattern of specialization inherent in GVCs can lock an economy into a carbon-intensive industrial structure, thereby elevating its aggregate carbon intensity. This observation is consistent with the classical articulation of scale and composition effects.^23^ As integration deepens, however, deeper participation in GVCs enhances the carbon reduction effect of IRs because it facilitates access to advanced environmental technologies and management expertise, improving the green production efficiency. Robots optimize energy and resource use with high precision, reducing carbon emissions during manufacturing. Moreover, deeper GVC involvement often entails stricter international environmental standards and competitive pressure, incentivizing firms to adopt cleaner technologies and further amplifying the decarbonization impact of robots. This dynamic pathway—from initial environmental pressures toward technology-driven green upgrading—is further corroborated by empirical evidence showing a non-linear relationship between GVC participation and carbon emissions.^24^

**Table 9:** Moderating effect of the degree of GVC participation

Variables	(1)	(2)	(3)
	lnAEI	lnAEI	lnAEI
lnRobot	−0.002∗∗∗	−0.001∗∗	0.018∗∗∗
	(-2.87)	(-1.99)	(4.03)
lnGVC_par	–	0.391∗∗∗	0.364∗∗∗
	–	(19.78)	(18.70)
lnRobot×lnGVC_par	–	–	−0.005∗∗∗
	–	–	(-4.42)
CONTROL	YES	YES	YES
FE	YES	YES	YES
Observations	16,716	16,664	16,664
R^2^	0.796	0.806	0.806

#### Moderating effect of the pattern of GVC participation

Within the value-added decomposition framework for trade, a country’s GVC participation is measured as the sum of its forward participation and backward participation. These metrics capture distinct patterns of integration into the GVC and, crucially, map onto different governance structures. Forward participation often aligns with positions in producer-driven chains or high-value-added segments of modular networks where technological capabilities and innovation incentives are stronger. Backward participation frequently corresponds to assembly roles in buyer-driven chains or captive supplier positions in highly asymmetric governance relationships.

Moreover, different participation patterns have different effects on the economic development and CEI. First, backward GVC participation tends to represent low-value-added and high-carbon production at the middle end of the “smile curve,” often associated with captive governance structures where suppliers have limited autonomy. So, the more backward the GVC participation is, the more energy-intensive activities are there, and the more are the carbon emissions.^25^ Second, forward GVC participation can occur in two main ways: producing high-quality intermediate goods for export, which is typical of producer-driven chains and advanced modular networks, or exporting raw materials such as fossil fuels. Developed countries usually participate in the former channel, while developing countries participate in the latter channel, and both channels are conducive to improving environmental quality and low-carbon development.^26^ Columns (2) and (4) of Table 10 reveal the divergent effects of GVC integration on the CEI. The coefficient for forward participation (ln*GVC_f*) is significantly negative (−0.067), indicating that upstream integration, which often reflects participation in technology-intensive producer-driven chains, is associated with a lower CEI. Conversely, backward participation (ln*GVC_b*) has a significantly positive coefficient (0.487), indicating that downstream integration, which frequently corresponds to the assembly roles in buyer-driven or captive chains, is associated with a higher CEI. The opposing effects stem from fundamentally different innovation incentives in GVCs: forward participation may contribute to a lower CEI by unlocking technology spillovers from advanced economies, enabling structural upgrading toward R&D-intensive activities and harnessing the scale efficiency gains, which are the dynamics more prevalent in the producer-driven and modular governance contexts. Conversely, backward participation increases the CEI by locking economies into pollution haven traps, where imported intermediates embed carbon-intensive processes and cost-competitive pressures suppress green innovation, which is a pattern characteristic of captive governance relationships in buyer-driven chains.

**Table 10:** Moderating effect of the GVC participation pattern

Variables	(1)	(2)	(3)	(4)	(5)
	lnAEI	lnAEI	lnAEI	lnAEI	lnAEI
lnRobot	−0.002∗∗∗	−0.002∗∗∗	0.006∗∗∗	−0.001	0.003
	(-2.87)	(-2.62)	(3.15)	(-1.04)	(1.17)
lnGVC_f	–	−0.067∗∗∗	−0.084∗∗∗	–	–
	–	(-7.87)	(-9.49)	–	–
lnRobot×lnGVC_f	–	–	−0.003∗∗∗	–	–
	–	–	(-4.40)	–	–
lnGVC_b	–	–	–	0.487∗∗∗	0.481∗∗∗
	–	–	–	(34.24)	(33.65)
lnRobot×lnGVC_b	–	–	–	–	−0.001
	–	–	–	–	(-1.50)
CONTROL	YES	YES	YES	YES	YES
FE	YES	YES	YES	YES	YES
Observations	16,716	16,556	16,556	16,664	16,664
R^2^	0.796	0.799	0.799	0.828	0.828

As shown in Table 10, the interaction term (ln*Robot* × ln*GVC_f*) in column (3) is significantly negative, indicating that the degree of forward-linkage GVC participation has a negative moderating effect on the relationship between IRs and CEI. In other words, higher forward GVC participation strengthens the carbon reduction effect of IRs, which may be due to the enhanced absorption of energy-efficient automation technologies and compliance with stringent environmental standards in advanced markets, which are the dynamics more pronounced in producer-driven governance structures where lead firms actively coordinate technology transfer. The interaction term (ln*Robot* × ln*GVC_b*) in column (5) is not significant, suggesting that the carbon emission reduction effect of robots is not affected by the degree of participation in backward-linkage GVCs. This non-significance may reflect the constraining effects of captive governance relationships, where suppliers’ limited decision-making autonomy restricts their capacity to leverage automation for emission reduction.^27^ As the degree of the country’s forward-linkage GVC participation increases, the country’s industrial structure tends to become more advanced, along with technological development as well as changes in the social environment, such as institutional norms, which provide a favorable development basis for the upgrading of robots, the expansion of the scale, and the installation of ancillary facilities. Hence, increased forward participation, which is often associated with movement toward producer-driven or high-value modular governance, amplifies the inhibitory effect of IRs on the CEI. With relatively high levels of backward-linkage GVC participation and comparative advantages in labor or natural resources, countries downstream to GVCs are mostly involved in low-end manufacturing within captive governance relationships and may experience a “low-end lock-in effect,” which may undermine effective carbon reduction through automation.

#### Moderating effect of GVC position

The development of capital- and technology-intensive sectors will be prioritized as the country’s GVC position advances. It is important to acknowledge that a high GVC position inherently confers structural advantages, including greater access to capital, stricter internal and external environmental regulation pressures, and stronger technological foundation—all of which contribute to better environmental performance independent of IR application. The expansion of high-value-added sectors serves as both a foundation and a driving force for widespread IRs, which may help unlock their potential in lowering the CEI. Thus, improving the GVC position may enhance the impact of IRs on reducing the CEI.

Notably, this study goes beyond merely observing that “high GVC position correlates with better emission performance” to reveal the “how and why”: IR technology acts as a specific, measurable transmission channel that empowers and transforms these inherent structural advantages into tangible environmental performance. The contribution of this study does not lie in discovering that a high-GVC-position enterprises perform better environmentally but in identifying the unique mediating role of IRs in this process. Specifically, we clearly distinguish between GVC structural advantages and the IR mechanism: enterprises with a high GVC position do possess inherent advantages, but IRs serve as an additional, independent carbon reduction channel beyond these advantages. After controlling for factors such as firm size, capital intensity, and regulatory pressure in our regression model, IRs still exert a significant and independent carbon reduction effect.

As listed in column (2) of Table 11, the coefficient of the GVC position index (ln*GVC_pos*) is significantly negative at approximately −0.352, suggesting that an increase in the GVC position contributes to a reduction in the CEI, which is consistent with the previous analysis. The regression results in Table 11 (column (3)) reveal a significant negative coefficient for the interaction term (ln*Robot* × ln*GVC_pos*), which indicates that after controlling for GVC structural heterogeneities, IRs still exhibit an independent carbon reduction effect, and this effect is stronger in GVC chains with high governance standards and high power positions. A higher position in GVCs typically involves technology-intensive activities, enhancing the capacity to adopt and innovate with clean production technologies. Such positions are also subject to stricter environmental standards and green trade barriers, motivating firms to leverage robotics for low-carbon transformation. Furthermore, advanced specialization is often coupled with greater digitalization and smart manufacturing, which amplifies the energy-saving and emission reduction potential of IRs. In summary, hypothesis 3 is verified.

**Table 11:** Moderating effect of GVC position

Variables	(1)	(2)	(3)
	lnAEI	lnAEI	lnAEI
lnRobot	−0.002∗∗∗	−0.002∗∗∗	−0.002∗∗∗
	(-2.87)	(-2.97)	(-3.40)
lnGVC_pos	–	−0.352∗∗∗	−0.414∗∗∗
	–	(-6.70)	(-7.52)
lnRobot×lnGVC_pos	–	–	−0.011∗∗∗
	–	–	(-2.70)
CONTROL	YES	YES	YES
FE	YES	YES	YES
Observations	16,716	16,716	16,716
R^2^	0.796	0.797	0.797

### Policy recommendations

First, for lead firms and high-position countries within GVCs, policy should encourage the exercise of technological advantages and governance power to fulfill environmental responsibilities as chain leaders. These firms possess the capability to establish and diffuse best practices in robot-enabled carbon reduction throughout their value chains. Specifically, policies could mandate or incentivize lead firms to integrate carbon footprint algorithms, green procurement standards, and clean production protocols into their supplier requirements. By leveraging their governance authority, lead firms can accelerate the adoption of energy-efficient automation technologies among downstream suppliers, thereby amplifying the systemic emission reduction impact beyond their own operational boundaries.

Second, for supplier firms and developing economies participating in GVCs, policy should prioritize enhancing the technological absorption capacity. Given the dual objective of GVC upgrading and green transition, targeted interventions could include tax incentives for robot adoption, preferential financing mechanisms, and technology extension services that reduce entry barriers to automation. Particular focus should be directed toward export-oriented sectors facing stringent environmental regulations, such as electronics and textiles, where compliance with international green standards is increasingly imperative for market access. These measures would enable supplier firms to leverage automation for both competitiveness enhancement and emission reduction, potentially facilitating movement toward higher value-added positions within GVC governance structures.

Third, for energy-intensive and capital-intensive industries, policy should emphasize the application of robots in process retrofitting and energy management systems that directly address emission bottlenecks. In sectors such as chemicals, basic metals, and non-metallic mineral products, where production processes are inherently carbon intensive, policies could support the integration of robotics with advanced process control, waste heat recovery systems, and real-time energy monitoring. Such applications target the fundamental drivers of emissions rather than peripheral activities, thereby maximizing the carbon reduction potential per unit of automation investment.

Fourth, for labor-intensive industries, policy could highlight the co-benefit of robot applications in improving occupational health and addressing labor shortages, alongside their synergistic carbon reduction effects. In sectors such as apparel, food processing, and electronics assembly, where workforce welfare and demographic transitions pose significant challenges, framing automation as a tool for both social and environmental objectives may garner broader societal support. Policies could include demonstration programs showcasing how robotic automation reduces workplace injuries, mitigates ergonomic risks, and simultaneously optimizes energy use in lighting, heating, and material handling systems.

Furthermore, policy should leverage the efficiency gains brought by robots as the legacy of Industry 4.0 to fund and enable the human-centric sustainability transition envisioned in Industry 5.0. This requires institutional mechanisms that channel productivity dividends from automation into investments in workforce reskilling, social protection systems, and circular economy infrastructure.

### Limitations of the study

The study has several limitations. First, it focuses exclusively on the association between operational IRs and carbon emission intensity without accounting for embedded emissions from production, installation, and energy consumption during use. This omission may lead to an overestimation of the net carbon reduction effect, and future research should address this data gap by incorporating upstream and operational flow data. Second, beyond the three macro-level channels examined, namely GTFP, energy consumption scale, and energy structure, micro-level mechanisms such as production process reengineering and supply chain coordination remain unexplored due to concerns over measurement feasibility and directness of impact. Firm-level microdata or case studies could open this black box in future investigations. Third, the empirical results capture only the average moderating effect of GVC participation across heterogeneous governance structures. This aggregation may mask divergent dynamics within categories like backward participation, for instance, differences between the modular and captive relationships, thus calling for more granular and context-specific analyses.

## Resource availability

### Lead contact

Requests for further information, resources, and reagents should be directed to and will be fulfilled by the lead contact, Genqiang Li (lgqwhu@163.com).

### Materials availability

This work did not generate new unique reagents. All materials and methods used for data generation and analysis are mentioned in the article.

### Data and code availability

• The data used in this paper are publicly available and are listed in the key resources table with accessibility.
• The code will be shared by the lead contact upon request.
• Any additional information required to reanalyze the data reported in this article is available from the lead contact upon request.

## Acknowledgments

This work was supported by the Humanities and Social Science Foundation of 10.13039/501100002338Ministry of Education of China (23YJC790058) and the 10.13039/501100012226Fundamental Research Funds for the Central Universities (501WKPY2026111004).

## Author contributions

Conceptualization, G.L.; methodology, G.L. and L.K.; software, L.K.; validation, G.L.; formal analysis, G.L.; investigation, G.L. and L.K.; data curation, G.L.; writing – original draft, L.K.; writing – review & editing, G.L. and L.K.; visualization, L.K.; supervision, project administration, and funding acquisition, G.L.

## Declaration of interests

The authors declare that they have no known competing financial interests or personal relationships that could have appeared to influence the work reported in this paper.

## Declaration of generative AI and AI-assisted technologies in the writing process

No generative AI or AI-assisted technologies were used in the manuscript preparation process.

## STAR★Methods

### Key resources table

**Table:** 

REAGENT or RESOURCE	SOURCE	IDENTIFIER
**Deposited data**		

Carbon Emission Intensity (CEI)	World Input-Output Database (WIOD)–Environmental Accounts	http://www.wiod.org/
Industrial Robots (IRs)	International Federation of Robotics (IFR)–World Robotics Industrial Robots	https://ifr.org
Degree of GVC Participation (GVC_par)	UIBE Global Value Chain Indicators Database	http://gvcdb.uibe.edu.cn/gvc.html
Forward Linkage GVC Participation (GVC_f)	UIBE Global Value Chain Indicators Database	http://gvcdb.uibe.edu.cn/gvc.html
Backward Linkage GVC Participation (GVC_b)	UIBE Global Value Chain Indicators Database	http://gvcdb.uibe.edu.cn/gvc.html
GVC Position (GVC_pos)	UIBE Global Value Chain Indicators Database	http://gvcdb.uibe.edu.cn/gvc.html
Green Total Factor Productivity (GTFP)	Calculated based on WIOD data	http://www.wiod.org
Energy Structure (struct)	WIOD Environmental Accounts	http://www.wiod.org
Energy Consumption Scale (pec)	WIOD Environmental Accounts	http://www.wiod.org
Urbanization Level (urb)	World Bank-World Development Indicators (WDI)	https://data.worldbank.org
Capital–Labor Ratio (kl)	Calculated based on WIOD data	http://www.wiod.org
Industry-Level Value Added per Capita (pgo)	Calculated based on WIOD data	http://www.wiod.org
R&D Intensity (RD)	World Bank-World Development Indicators (WDI)	https://data.worldbank.org

**Software and algorithms**		

Stata/MP	StataCorp LLC	https://www.stata.com

### Method details

#### Theoretical analysis and research hypotheses

The theoretical framework diagram of this study is shown in the figure above.

**Figure:**
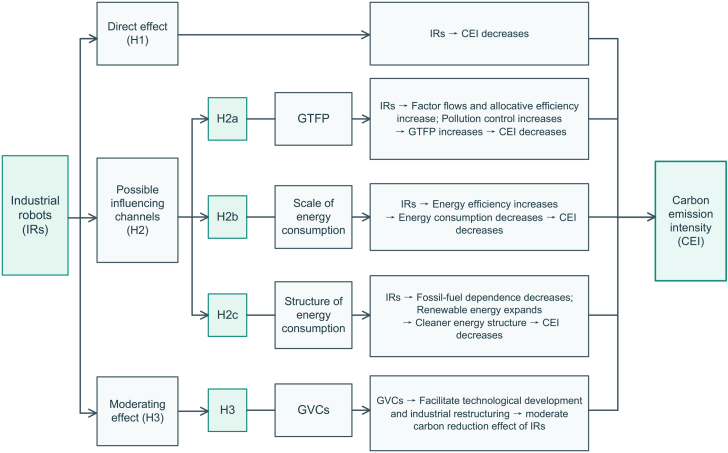
Theoretical framework diagram

The environmental Kuznets curve (EKC) indicates an inverted-U correlation between economic development levels and environmental quality. From a technological perspective, accelerated economic growth is associated with intensive R&D investment. In the course of a nation's economic development, an increase in R&D spending will foster technological advancement, including the use of IRs, which will increase the effectiveness of resource and energy use, lower resource inputs per unit of output, lower carbon emissions per unit of output, and generally increase environmental standards. By using robots, it is possible to achieve the regeneration of straw fermentation while also improving the accuracy of solid pollutants compared with artificial methods, which lower the intensity of carbon emissions.^28^ The industrial structure also changes as a result of economic growth and technological advancement, moving from the industrial economy's high energy consumption to knowledge- and technology-based economies. This transition alleviates resource misallocation, facilitates the rational distribution of energy resources, thereby effectively improving energy utilization efficiency, fostering the application of energy-saving technologies, and reducing fossil fuel consumption.^29^ Furthermore, technological progress enhances firm productivity, thereby attracting foreign direct investment (FDI). On one hand, it facilitates industrial upgrading and the energy transition through technology spillovers; on the other hand, it motivates enterprises to innovate production technologies to reduce fossil fuel consumption and improve environmental benefits.^30^^,^^31^ Therefore, this paper proposes the following hypothesis:

H1: IRs reduce CEI.

As a core embodiment of digital and automation technologies, IRs drive green growth and CEI reduction by promoting green total factor productivity (GTFP) growth, which aligns with environmental endogenous growth theory and the “Green Solow Model”—the latter extends traditional growth frameworks by incorporating pollution and abatement, and posits that long-term environmental sustainability depends on GTFP growth.^32^ This GTFP-driven pathway is realized through two synergistic dimensions. On the input side, IRs promote the reallocation of production factors (human capital, physical capital, and natural resources) from labor- and capital-intensive industries to technology-intensive ones, thereby improving factor flow efficiency and allocation rationality.^33^ On the output side, IRs’ powerful data collection and processing capabilities enable real-time monitoring of production environments and precise control of pollution emissions; meanwhile, they effectively mobilize employee participation in R&D, streamlining the development of green technologies and optimizing green products, which comprehensively enhances green innovation capacity.^34^ These two aspects jointly drive dynamic GTFP growth, providing a fundamental driving force for CEI reduction. Accordingly, this paper proposes the following hypothesis:

H2a: IRs reduce CEI by increasing GTFP.

Guided by ecological economics and energy transition theory (a systematic theoretical framework that examines the long-term, structural transformation of energy systems from fossil fuel dominance toward low-carbon and sustainable configurations), IRs further reduce CEI by regulating the scale and structure of energy consumption—a key pathway complementing the GTFP channel. According to ecological economics, the fossil fuels used to support economic growth will not vanish but will eventually return to the ecosystem in the form of waste, which will have a detrimental effect on the ecosystem, such as the specific manifestation of rising carbon emissions. Thus, technological advancement (including IRs) to promote green energy use has become a core strategy for low-carbon transformation. More specifically, there are two aspects of the impacts on energy consumption: First, in terms of energy consumption scale, energy efficiency can be enhanced through reduced energy input per unit of output.^35^^,^^36^ While classical energy economics highlights the “rebound effect” (efficiency gains may increase energy demand by lowering energy service costs, offsetting savings), IRs—representing intelligent technological progress—can transcend this limitation. Through precise control and systemic optimization of production processes, IRs minimize redundant energy use, achieving “super-conservation” that not only reduces unit output energy input but also induces structural reductions in absolute energy demand, potentially overcoming the traditional rebound effect.^37^ Additionally, IRs promote the installation of supporting infrastructure, facilitating the replacement of high-carbon equipment and achieving comprehensive CEI reduction. Second, in terms of energy consumption structure, IRs promote the transition toward cleaner energy, which aligns with the core tenet of “energy transition” theory that deep decarbonization relies on energy system transformation.^38^ IRs enhance energy efficiency through green technology innovation and drive the development of alternative clean energy, thereby optimizing the energy consumption structure.^39^ From the demand side, IRs are mainly powered by electricity, reducing the use of traditional energy; from the supply side, IRs support the expansion of renewable energy industries and the decline of traditional energy sectors. This strengthens the substitution effect of renewable energy, reduces fossil fuel dependence, and promotes energy structure transition, further reducing CEI.^40^ In summary, this paper proposes the following hypotheses:

H2b: IRs reduce CEI by reducing the scale of energy consumption.

H2c: IRs reduce CEI by optimizing the structure of energy consumption.

GVC participation plays a critical role in shaping the carbon reduction effect of IRs. Integrating classical GVC governance theory, we recognize that the nature of this moderating effect is contingent upon the governance structure of the value chain. Building on the seminal GVC framework, Gereffi’s foundational typology differentiates producer-driven chains—represented by capital-intensive sectors including automobiles, machinery, and electronics—from buyer-driven chains concentrated in labor-intensive industries such as apparel, footwear, and consumer goods.^41^ Subsequent research has extended this perspective to examine industrial upgrading, regional embeddedness, and supplier dynamics within transnational production networks.^10^^,^^11^ Meanwhile, incomplete contract models have endogenized GVC organizational structures, clarifying how institutional and contractual frictions shape cross-border production fragmentation and value chain governance.^8^ In producer-driven GVCs, characterized by high technological intensity, significant capital requirements, and strong lead firm coordination, the carbon reduction potential of IRs is likely amplified. These chains offer greater opportunities for technology spillovers, process upgrading, and the integration of energy-efficient automation. Conversely, in buyer-driven GVCs, where price sensitivity and low-cost labor arbitrage dominate, the “low-end lock-in” effect may suppress green innovation, potentially weakening the emissions-reducing impact of automation.^42^ Furthermore, governance patterns involving high power asymmetry, such as those found in captive networks, may limit suppliers' capacity to invest in cleaner technologies, whereas more symmetric relationships, for example under modular governance, could facilitate the diffusion of green automation.^43^ It is important to clarify that the empirical results presented in this paper represent the average effect across diverse GVC types and governance structures. In producer-driven chains with high technological integration and coordinated lead firm relationships, the carbon reduction effect of robots is likely stronger than our average estimates suggest, whereas in buyer-driven chains with captive governance structures, the effect may be considerably weaker.

Overall, GVC participation can play a role in the carbon reduction effect of IRs. On the one hand, GVC participation facilitates the global dissemination of technological resources, accelerating growth in technology/knowledge-intensive clean industries and advancing digital technologies such as industrial robots—thereby enhancing their carbon reduction potential. On the other hand, it hinders the development of IRs and the establishment of a massive industrial chain, placing a country's industrialization process in a “low-end lock-in” situation and causing it to become a “pollution shelter”. Therefore, the country may experience miserable growth.^44^ As a result, when a country’s degree of GVC participation or GVC position is low, it is difficult to adjust the industrial structure of that country, which then remains in high-energy and high-polluting industries, inhibiting IRs from lessening CEI. This paper advances the following hypothesis:

H3: The degree of GVC participation, the GVC participation pattern, and the GVC position moderate the effect of IRs on reducing CEI.

#### Key variables

We measure carbon emission intensity (CEI) using the aggregate embodied intensity (AEI) approach proposed by Su and Ang.^45^^,^^46^ The AEI is calculated as the ratio of implied carbon emissions produced by each industry to the implied value added. Unlike traditional territorial-based emission intensity metrics, the AEI accounts for carbon emissions embodied in international trade by tracing emissions across the entire global production network. All upstream emissions generated throughout the supply chain are allocated to the final products consumed within a country. This characteristic makes the AEI particularly suitable for global value chain (GVC) contexts, as it attributes emissions from imported intermediates to the final demand of the consuming nation rather than the producing nation.

(Equation 1) $$AEI=\frac{E}{GDP}=\frac{f_{v}^{\prime }H_{d}y}{1^{\prime }H_{d}y}$$

where *E* denotes the total implied emissions from the production process, $f_{v}^{\prime }$ denotes the emissions per unit of value added, *H*_*d*_ is the matrix of domestic demand coefficients for value added, and y is the domestic final demand; the *GDP* (domestic value added) comprises implied value added from domestic final consumption and exports.

To capture the moderating role of global value chain integration, we construct three representative GVC indicators following established methodologies. First, the degree of GVC participation (GVC_par) is measured by summing forward and backward linkage participation as in Borin and Mancini.^47^ Second, forward-linkage GVC participation (GVC_f) captures the share of domestic value added embodied in intermediate exports relative to total exports. Backward-linkage GVC participation (GVC_b) measures the ratio of foreign value added in imported intermediates to final goods output. Formal definitions are given in Equations 2, 3, and 4. Third, the GVC position (GVC_pos) is derived following Wang et al.^48^ This indicator measures the distance between a country or sector and all relevant producing countries or sectors, calculated as the difference between forward production length and backward production length, normalized by total production length, as shown in Equation 5. A higher GVC_pos indicates a more upstream position in the value chain. All GVC indicators are obtained from the UIBE GVC database, which provides harmonized estimates across countries and industries.

(Equation 2) GVC_par=GVC_f+GVC_b

(Equation 3) $$GVC\_f=\frac{V_{s}{\left(I-A_{ss}\right)}^{-1}A_{sr}{\left(I-A_{rr}\right)}^{-1}\left(\sum_{j\neq r}^{G}Y_{rj}+\sum_{j\neq r}^{G}A_{rj}\sum_{k}^{G}\sum_{l\neq s}^{G}B_{jk}Y_{kl}\right)}{u_{N}E_{sr}}$$

(Equation 4) $$GVC\_b=\frac{V_{s}{\left(I-A_{ss}\right)}^{-1}\sum_{j\neq s}^{G}A_{sj}B_{js}E_{sr}+\sum_{t\neq s}^{G}V_{t}B_{ts}E_{sr}}{u_{N}E_{sr}}$$

(Equation 5) $$GVC\_pos=\frac{PLv\_GVC}{{\left[PLy\_GVC\right]}^{\prime }}=\frac{Xv\_GVC/V\_GVC}{Xy\_GVC/Y\_GVC}$$

#### Data

The data are primarily drawn from four sources. IR data is obtained from the International Federation of Robotics (IFR). Carbon emission data and input–output tables are from the latest World Input–Output Database (WIOD). However, although other input–output databases have updated data, none of them provide country-specific, industry-specific data on carbon emissions. Thus, the WIOD database used in this paper is the most recent database available that includes both input–output linkages and carbon emissions. To eliminate the influence of the price factor, this paper adjusts the variables with the output price index and takes 2010 as the base period. The R&D investment and urbanization level data are obtained from the World Bank (WDI). The UIBE GVC database is the source of the degree of participation, pattern and position of GVC .

In this paper, the industries are matched on the basis of the industry name in accordance with the practice of Wang et al.^49^ to ensure the consistency of the statistical caliber because of differences between the IFR industry classification and the WIOD industry classification standards. Because of data availability, Taiwan and the rest of the world are excluded, and the data of 56 industries in 42 economies are selected for measurement. This volume of information is significant on a broad scale since it may be used to represent the great majority of representative economies and industries. All continuous variables were winsorized at the 1st and 99th percentiles to reduce the influence of outliers. Following the methods of Wooldridge et al.,^50^ absolute-valued variables are log-transformed to mitigate skewness. This process compresses the scale of the variables and attenuates the problem of heteroskedasticity of the model while not changing the relative relationship of the data. Owing to the presence of some data of 0 for both the *Robot* and the *AEI* variables, this paper takes the logarithm of the two variables after adding 0.0001.

#### High-dimensional fixed-effect model

To examine the impact of industrial robots on carbon emission intensity, we estimate a benchmark regression model with high-dimensional fixed effects. The specification is as follows:

(Equation 6) $$\ln\;AEI_{i,j,t}=\alpha +\alpha_{1}\;\ln\;Robot_{i,j,t}+\sum_{k=2}^{5}\alpha_{k}\;\ln\;X_{i,j,t}+\delta_{i}+\mu_{j}+\gamma_{t}+\varepsilon_{i,j,t}$$

where *AEI*_*i*,*j*,*t*_ is the CEI and *R*obot_*i*,*j*,*t*_ represents the scale of industrial robot utilization. *X*_*i*,*j*,*t*_ is a vector of control variables consisting of industry–level value added per capita, the capital–labor ratio, the urbanization level, and research and development intensity. *δ*_*i*_,*μ*_*j*_,*γ*_*t*_ denote country, industry, and year fixed effects (FE), respectively. *ε*_i,*j*,*t*_ is the idiosyncratic error term, with subscripts i (country), j (industry), and t (year). This high-dimensional fixed-effects structure enables us to control for unobserved heterogeneity across both countries and industries over time, thereby isolating the causal effect of industrial robots on carbon emission intensity.

#### Mediation effect model

We adopt the three-step mediation procedure proposed by Wen et al.,^51^ which is widely used in empirical economics. The approach tests whether the effect of industrial robots on carbon emission intensity operates through intermediate channels, including green total factor productivity (GTFP), energy consumption scale (pec), and energy structure (struct). The three equations are specified as follows:

(Equation 7) $$\ln\;AEI_{i,j,t}=\alpha +\alpha_{1}\;\ln\;Robot_{i,j,t}+\sum_{k=2}^{5}\alpha_{k}\;\ln\;X_{i,j,t}+\delta_{i}+\mu_{j}+\gamma_{t}+\varepsilon_{i,j,t}$$

(Equation 8) $$M_{i,j,t}=\alpha +\alpha_{1}\;\ln\;Robot_{i,j,t}+\sum_{k=2}^{5}\alpha_{k}\;\ln\;X_{i,j,t}+\delta_{i}+\mu_{j}+\gamma_{t}+\varepsilon_{i,j,t}$$

(Equation 9) $$\ln\;AEI_{i,j,t}=\alpha +\alpha_{1}\;\ln\;Robot_{i,j,t}+\alpha_{2}M_{i,j,t}+\sum_{k=3}^{6}\alpha_{k}\;\ln\;X_{i,j,t}+\delta_{i}+\mu_{j}+\gamma_{t}+\varepsilon_{i,j,t}$$

Equation 7 is the baseline model, the dependent variable *M* in Equation 8 represents the mediator variables. The mediator variable *M* is added to Equation 9, the dependent variable is still CEI, and the explanatory and control variables remain unchanged.

#### Moderation effect model

To examine whether global value chain integration moderates the relationship between industrial robots and carbon emission intensity, we introduce interaction terms between the core independent variable (IRs) and each GVC indicator. The moderation model is specified as:

(Equation 10) $$\ln\;AEI_{i,j,t}=\alpha +\alpha_{1}\;\ln\;Robot_{i,j,t}+\alpha_{2}Z_{i,j,t}+\alpha_{3}\;\ln\;Robot_{i,j,t}\times Z_{i,j,t}+\sum_{k=4}^{7}\alpha_{k}\;\ln\;X_{i,j,t}+\delta_{i}+\mu_{j}+\gamma_{t}+\varepsilon_{i,j,t}$$

where *Z*_*i*,*j*,*t*_ represents the moderating variable (GVC_par, GVC_f, GVC_b, or GVC_pos). To reduce multicollinearity and facilitate interpretation, we mean-center the IRs and GVC variables before constructing the interaction term. The coefficient *α*_3_ captures the moderating effect: a significant negative *α*_3_ indicates that stronger GVC participation enhances the carbon reduction effect of industrial robots, whereas a significant positive *α*_3_ suggests a dampening influence.

### Quantification and statistical analysis

We conducted the data analysis using StataMP-64 software. For all continuous variables in the model, we winsorized them at the 1st and 99th percentiles, and performed logarithmic transformation on the absolute value variables.^50^ Descriptive statistics show the number of variables, their average values, standard deviations, minimum values, median values, and maximum values.

In addition to the above procedures, we conducted benchmark regression analysis, mediation mechanism analysis, and moderation effect analysis using StataMP-64. For each estimation, we reported the regression coefficients of the independent variable and control variables, the associated t-values, the fixed effects included in the model to control for unobserved heterogeneity, and the R-squared values as measures of overall model fit. These analyses collectively enabled us to examine the direct carbon reduction effect of industrial robots, the underlying transmission channels, and the moderating role of global value chain participation.

## Supplemental information

Data S1. Processed data and analysis code supporting the empirical analysis of industrial robots, carbon emission intensity, and global value chains
